# Changing the firing threshold for normal optic nerve axons by the application of infra-red laser light

**DOI:** 10.1038/s41598-021-00084-1

**Published:** 2021-10-15

**Authors:** Lavinia J. Austerschmidt, Nadine I. Schottler, Alyssa M. Miller, Mark D. Baker

**Affiliations:** 1 Centre for Neuroscience, Surgery and Trauma, Blizard Institute, QMUL 4 Newark Street Whitechapel, London, E1 2AT UK; 2 Present Address: Yusuf Hamied Department of Chemistry, University of Cambridge, Lensfield Road, Cambridge, CB2 1EW UK

**Keywords:** Biophysics, Biotechnology, Neuroscience, Physiology

## Abstract

Normal optic nerve axons exhibit a temperature dependence, previously explained by a membrane potential hyperpolarization on warming. We now report that near infra-red laser light, delivered via a fibre optic light guide, also affects axonal membrane potential and threshold, at least partly through a photo-thermal effect. Application of light to optic nerve, at the recording site, gave rise to a local membrane potential hyperpolarization over a period of about a minute, and increased the size of the depolarizing after potential. Application near the site of electrical stimulation reversibly raised current-threshold, and the change in threshold recorded over minutes of irradiation was significantly increased by the application of the *I*_h_ blocker, ZD7288 (50 µM), indicating *I*_h_ limits the hyperpolarizing effect of light. Light application also had fast effects on nerve behaviour, increasing threshold without appreciable delay (within seconds), probably by a mechanism independent of kinetically fast K^+^ channels and Na^+^ channel inactivation, and hypothesized to be caused by reversible changes in myelin function.

Several lines of evidence suggest that optic nerve axon membrane potentials must be temperature-dependent to a greater degree than that predicted from the effect of temperature on ionic reversal potentials^1–3^. In order to study further this phenomenon we have investigated the effects of local near infra-red (IR) laser-light irradiation applied to optic nerve (with a wavelength of 1550 nm) via a light guide, and the reason we did this is that the technique may offer a way to make more rapid temperature changes than those provided by changes of whole nerve bath perfusion temperature, and to provide a means of applying temperature changes only close to sites of particular interest, such as the site of stimulation and recording. One significant advantage of being able to change temperature locally, at the site of stimulation for example, is that no corresponding changes to the kinetics of the action potential or excitability of the nerve fibres recorded several millimetres away are expected, and, when recording current-threshold, changes in nerve behaviour cannot be confounded by changes in the properties of the recorded action potential.

We have previously reported the appearance of a period of superexcitability following an action potential with whole-nerve bath warming from near room temperature, and the disappearance of accommodation to long depolarizing currents found in a cool nerve, on warming. Both these observations are consistent with increasing membrane potential. Although recording membrane potential changes directly is problematic, we have been able to estimate the resting membrane potential temperature dependence in the optic nerve is up to − 2 mV °C^−1^ at physiological temperatures, in the fastest conducting F-fibres, on the basis of threshold changes^3^. We have argued that such changes in membrane potential may contribute to impulse propagation failure with a raised core body temperature, in axons with defects in conduction, because they reduce excitability, and this may help explain the extraordinary temperature dependence of symptoms in multiple sclerosis (MS).

Our key hypothesis put forward to explain the effect is the presence of temperature-dependent electroneutral Na^+^ movements across the membrane from outside to inside, coupled with a variable steady-state electrogenic component of the membrane potential generated by the active removal of Na^+^^1–3^. This possibility means that the electrogenic component to the membrane potential, under normal circumstances, must be larger than predicted from the influx of Na^+^ through ion channels alone. It also means that transport mechanisms bringing Na^+^ into axons, with a pharmacology distinct from that of Na^+^ channels, may be able to provide a rationale for the development of drugs that can modify excitability without interfering with impulse generation directly. To investigate the effect of temperature on the membrane potential, we have previously controlled the temperature in the whole ex vivo nerve bath, by warming the perfusing buffer solution. However, the bath temperature can take minutes to reach a new equilibrium. As it turned out, our data are not consistent with the effects of laser light being a simple, rapid warming only.

Near IR light is expected to be absorbed by water or lipid^4^, with bond vibration changes as a result. In physiological experiments, near IR light has been used to modify the behaviour of excitable tissues and although the mechanisms were mysterious for years, it now appears most likely caused by changes in membrane properties. How this energy absorption by brief pulses of light energy may be converted to changes in excitability was addressed by a detailed study that revealed that laser light increased the membrane capacitance, giving rise to a depolarization and a complementary inward current, in untransfected *Xenopus* oocytes and HEK cells^5^. In the study by Shapiro et al.^5^, the laser light provided mJ of irradiation within milliseconds. In contrast, we have endeavoured to study reversible temperature changes brought about locally, using light from a commercially rated 5 mW laser diode, a far lower power output. In these present experiments, most of our findings are consistent with changes in local temperature being essential for the changes in excitability we see, a photo-thermal effect previously noted by Wells et al.^6^.

## Results

### Application of IR laser light at the recording site increases the DAP

**Figure 1:**
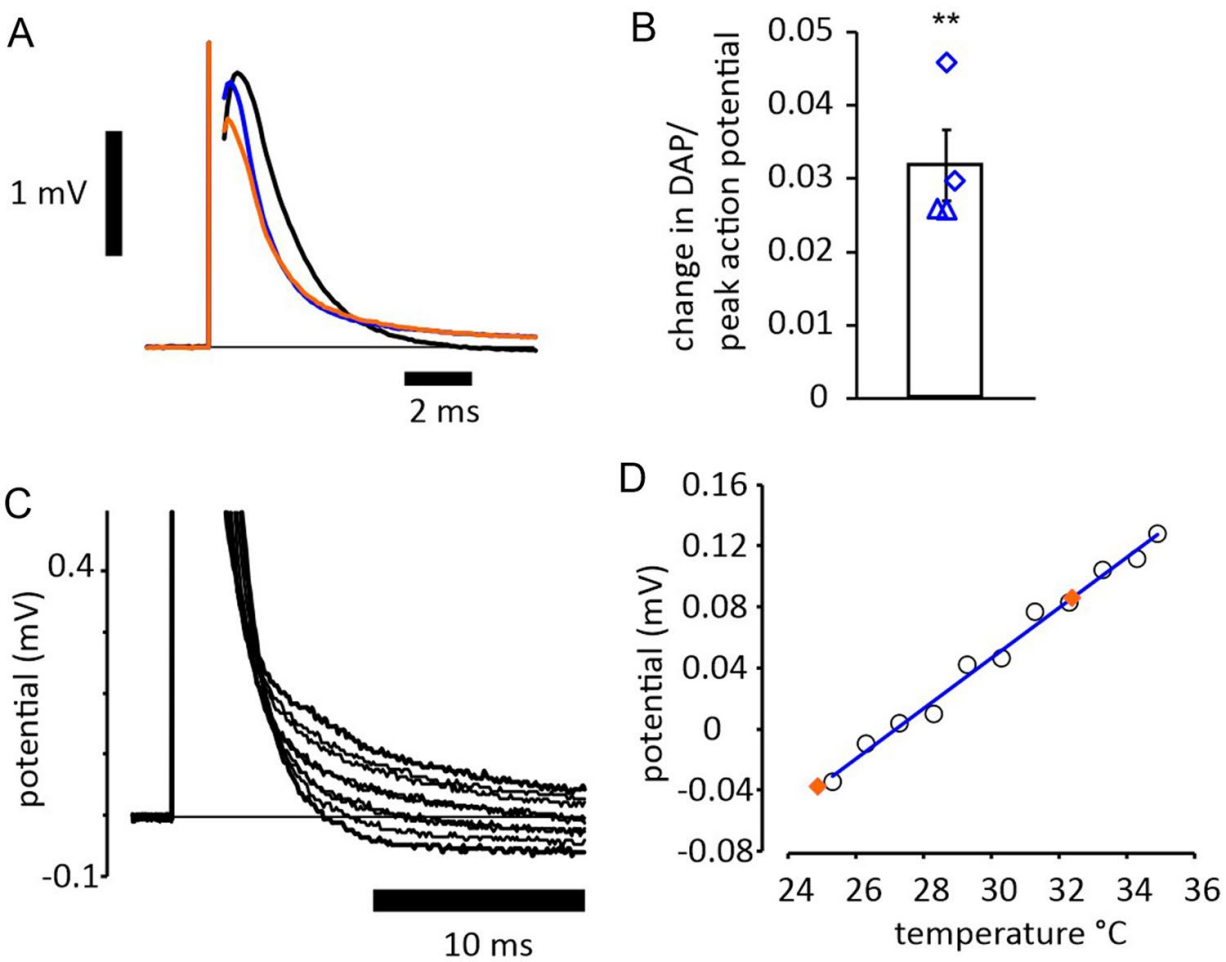
IR-laser light affects supramaximal evoked action potentials in a manner similar to whole-bath warming. (**A**) Control action potential evoked at a bath temperature of 25 °C (black trace), and at 32 °C (blue trace). With an ambient bath temperature of 25 °C, application of continuous laser light at the site of recording gives the third action potential (orange) within seconds, an effect sustained during the light application. The action potentials recorded either in the warmer bath, or with laser light application appear briefer, and have longer depolarizing afterpotentials (DAP). (**B**) A similar effect was observed in 4 nerves, with an increase in the DAP at 10 ms following stimulation near 3% of the peak action potential amplitude (*P* = 0.007, single-sample t-test, compared with 0), and not obviously affected by the presence of bumetanide and amiloride (5 µM and 10 µM, respectively), in the recording solution (control solution: blue diamonds; with Na^+^ transport blockers: blue triangles). For one nerve in control solution the change in the afterpotentials is shown in more detail in (**C**), with a gradually increasing ambient temperature from 25 to 35 °C and a monotonically increasing afterpotential. The relationship between DAP amplitude and ambient temperature, measured at 10 ms, is shown in (**D**), and superimposed are the measurements of DAP amplitude without and with laser light at an ambient temperature of 25 °C (orange diamonds). Simple comparison suggests the laser light produces a local warming of 7 °C in this nerve, although a part of the effect may be caused by changes in myelin function. As already reported, the changes in appearance of the action potential with warming are largely caused by changes in action potential kinetics, rather than conduction velocity.

The depolarizing afterpotential (DAP) in myelinated axons is generated by internodal charging from local active nodes^7^ and the amplitude and duration of the DAP depends on the internodal membrane conductance. This conductance is increased by depolarization, as internodal voltage-gated K^+^ channels activate^8^, and this makes the amplitude and the rate of decay of the DAP an indirect, although sensitive, index of membrane potential. Furthermore, our experiments with 4-aminopyridine (4-AP), see below, have confirmed that the DAP amplitude is controlled by 4-AP sensitive K^+^ channels, likely to be of the K_V_1 sub family see^9^, K_V_1.2. The amplitude of the DAP following a supramaximal compound action potential increased when laser light was applied with a 400 mA driving current (equivalent to 600 mJ per minute) (Fig. 1). Comparing the effects of applying laser light and the effects of altering whole-bath perfusion temperature, indicates that the laser diode when operating continuously, brings about local changes in temperature of a few single °C. As is also apparent in Fig. 1, the application of laser light differs subtly from raised perfusion temperature, in that it reliably caused a small drop in the amplitude of the compound action potential, that we understand is as a result of a rapid effect of laser light (discussed more fully below).

### Application of IR laser light at the site of nerve electrical stimulation makes optic nerve axons less excitable

**Figure 2:**
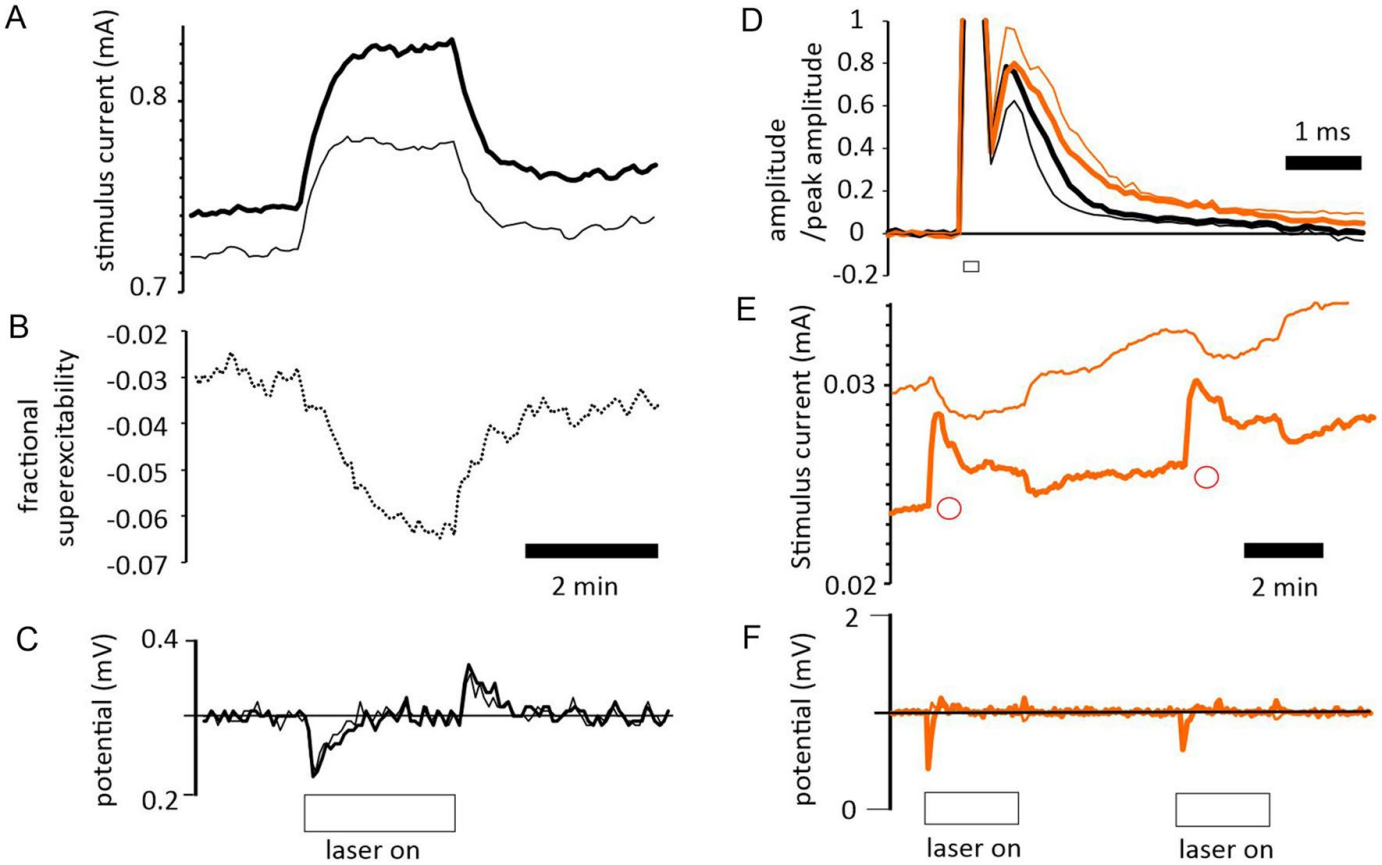
Application of IR laser light to a nerve at the site of stimulation, while threshold-tracking. (**A**) laser light application (indicated in panel (**C**), driving laser using 350 mA) gives rise to an increase in threshold when maintaining a 50% supramaximal response. Control threshold (bold trace), and threshold at 10 ms following a conditioning stimulus (fine trace) undergo increases. (**B**) The fractional difference between the control and conditioned threshold reveals increased superexcitability during the application of laser light. (**C**) The stimulus current used to generate the control (bold trace) and conditioned (fine trace) responses is unable to maintain response amplitude as soon as the light is activated, but over the course of 2 min increases enough to overcome the inhibition. When the light is removed, the threshold-tracking program brings the response amplitude down by reducing the stimulus current. (**D**) F-fibre response at 5% of supramaximal amplitude (black trace; mean, *n* = 4) is modified by exposure to 200 µM 4-AP (orange trace; mean, n = 4), with the action potential widening (at 0.5 amplitude, *P* = 0.02, paired t-test) and the depolarizing afterpotential (DAP) increasing in amplitude and duration. Application of stimulus current indicated as open bar, and means with − 1 sem (control) or + 1 sem (4-AP) are plotted; sems plotted as fine lines. Data collected in the presence of 4-AP plotted in orange. (**E**) Laser light application (using 400 mA driving current, ie 600 mJ per minute) indicated underneath panel (**F**), produces an immediate fall in the amplitude of the F-fibre response (see bold trace in (**F**) below), which is then compensated-for by a rise in the applied current, maintaining the target response amplitude. A rapid rise in threshold results, indicated by red circles, that is not fully maintained. The conditioned response threshold at 4 ms following a previous stimulus is a measure of refractoriness, and is plotted as a fine trace. Refractoriness falls during laser light application. (**F**) Changes in peak response amplitude during threshold-tracking, before, during and after laser light application. Bold and fine traces are responses to test and conditioned stimuli, respectively.

**Figure 3:**
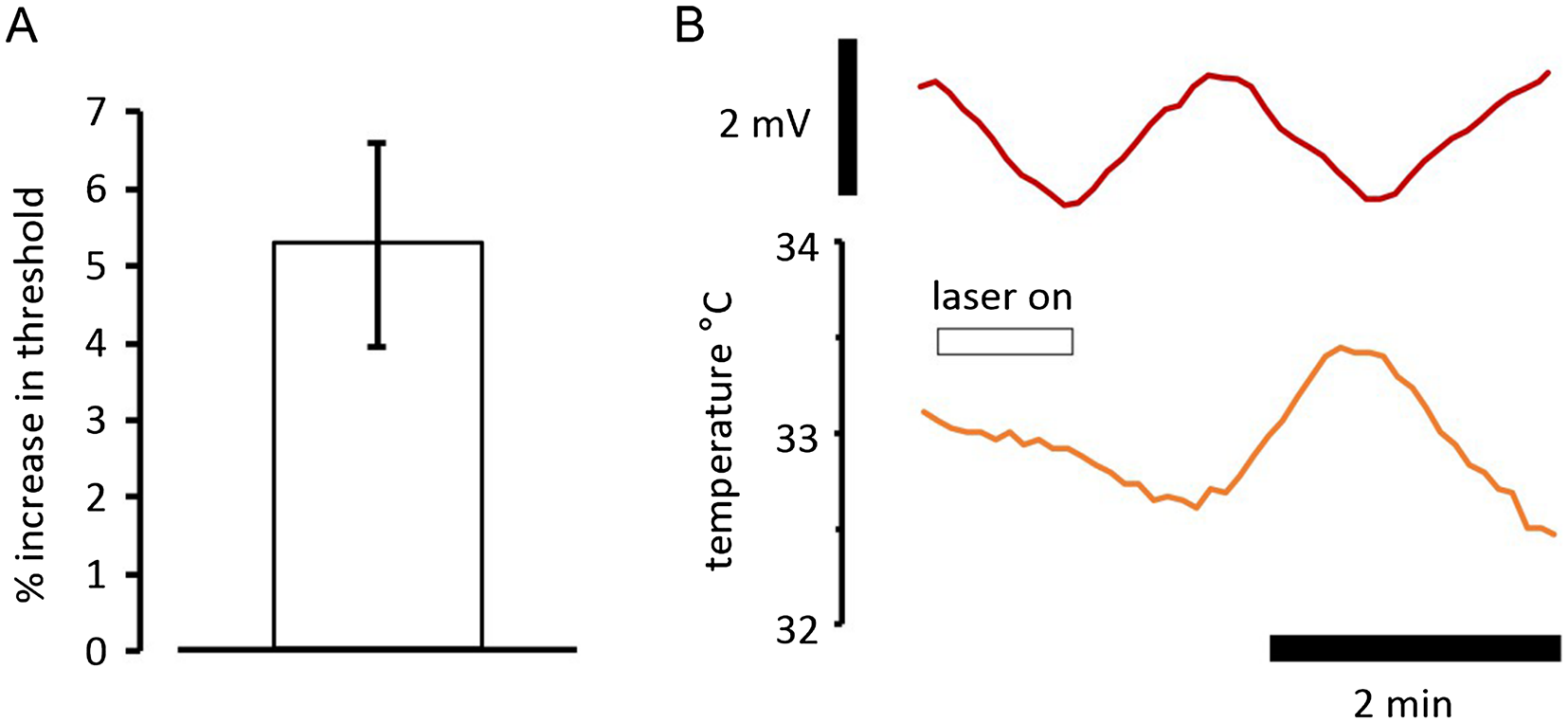
IR laser light application at the site of stimulation increases threshold; at the site of recording, the membrane potential hyperpolarizes. (**A**) With a driving current of 400 mA (equivalent to 600 mJ per minute), and continuous operation of the laser, a threshold increase is sustained for at least 3 min (*P* = 0.017, *n* = 5). (**B**) Extracellular membrane potential recording (upper panel) reveals relatively slow changes in potential on the application of laser light (in this case 350 mA driving current) that mimic membrane potential changes caused by ambient temperature fluctuations in the bath (lower panel). As these potentials are recorded extracellularly they must be smaller than the membrane potential changes found intra-axonally, the latter likely to be of the order of a few single mV. The sustained increase in threshold therefore appears to be caused by a temperature dependent membrane potential.

Switching on the IR laser irradiation at the stimulation site caused an immediate effect on the height of the recorded compound action potential. It was always reduced. Where the application of light was prolonged over minutes, the reduction in amplitude gradually lessened over this period, although it stayed reduced. Where a less than supramaximal action potential was evoked in the nerve, and the technique of threshold-tracking was employed to keep the response constant eg^3^, an increase in threshold was observed usually lasting throughout the duration of light application (Fig. 2A–C). Where the laser diode was powered at 400 mA, and with an ambient bath temperature of 32 °C the average increase in threshold measured at the end of 3 min applications was 5.26 ± 1.32% (mean ± sem, *P* = 0.017, one-sample t-test, *n* = 5), (Fig. 3A), consistent with an average local temperature change affecting the nerve close to 3 °C. This finding was anticipated from previously reported findings for changing bath ambient temperature^3^, and also supports the idea of a temperature dependent membrane potential. The small size of the temperature change revealed is the reason that the manoeuvre can be repeated without any noticeable acute damage to the axons.

The recordings in Fig. 2A and B provide clear evidence for an increase in superexcitability during the IR light application. The superexcitability trace shown in Fig. 2B was obtained by comparing the threshold measured in response to a control stimulus, with one preceded by a conditioning stimulus, 10 ms before. Superexcitability is a normal integral part of the recovery cycle following an action potential, and can be defined as a period during which axons become easier to excite following an action potential eg^10^. It can be contrasted with the refractory period that occurs closer to the response to the conditioning stimulus, during which threshold is increased. The recordings in Fig. 2E (and Supplementary Figure S1) show a fall in refractoriness during the application. The recorded increase in superexcitability at 10 ms, and fall in refractoriness at 4 ms, following a conditioning stimulus, occur gradually over the minute following the onset of light application in Fig. 2B, E, and their new level outlasts the period of less-than-adequate threshold-tracking occurring immediately after illumination (indicated by the fall in the peak response). This increase in superexcitability is to be expected from the effect of laser light on the DAP, and suggests that the nerve fibres hyperpolarize over this time. The fall in refractoriness implies Na^+^ channels are able to escape inactivation (reprime) more quickly during irradiation, and again suggests a hyperpolarization may be occurring. It therefore seems likely that a part of the reduction in excitability is caused by a warming induced membrane potential hyperpolarization, that we have already explored^1–3^. The most direct measurements of membrane potential changes with IR light application revealed that the warming effect is complete with a time course of a minute to minutes (eg Fig. 3B).

It is to be expected that near-IR light will be absorbed by water and lipid, both within and surrounding the isolated nerve, and because the nerve is constantly perfused in our nerve bath, heat energy will be continuously lost. We propose that the rate of axonal warming will depend critically on the properties of the interface between the fibre optic and the nerve, and this explains why the effect of exactly similar manoeuvres in different experiments can give variable results. We can also say that unless the end of the light guide is in physical contact with the nerve, no functional changes in the nerve are found, even if the light guide is very near the nerve when viewed under a stereo microscope, and so this contact is essential.

### IR laser light affects optic nerve axons rapidly

A consistent finding (during our recordings from F-fibres), is a rapid increase in threshold occurring without apparent delay on light application (Fig. 2C), and in other experiments a fall in the amplitude of a supra-maximal compound action potential. When recording supra-maximal compound action potentials, the whole of the action potential was affected, including the M and S components (data not shown), suggesting that this effect is not specific for any one nerve fibre category. Whether this fast increase in threshold can be seen when threshold-tracking depends critically on how quickly the tracking feedback-loop can compensate for changes in response amplitude. In the F-fibres, this rapid effect on threshold may not be sustained and may decay with continuous irradiation, whereas what we believe to be a hyperpolarization mediated increase in threshold probably remains, accounting for the overall trajectory of threshold in Fig. 2E (bold trace). This fast increase in threshold can also be seen as a brief upward shift in the threshold when the axons are in the relative refractory period measured at 4 ms after a conditioning stimulus, before changes in membrane potential reverse the direction of travel for the threshold-measurement (see Supplementary Figure S1). Also, the maximal change in threshold caused by the rapid effect is too large to be accommodated by the threshold-temperature relation we have already defined^2,3^. How might this rapid effect be explained? We can put forward two possibilities. The IR light may have direct effects on nodal ion channels, for example activating delayed-rectifier K^+^ channels^11^, or increasing Na^+^ channel inactivation. Na^+^ channel inactivation substantially impacts excitability^3,12^. A second possibility is that there is a rapid change in the properties of nodal membrane and/or trans-myelin (or Barrett and Barrett^7^) resistance acting to increase the effective nodal capacitance, and hence reduce excitability. However, our evidence suggests that an ion channel mechanism may not be tenable, because blocking K^+^ channels (Figs. 2D and S1) does not prevent the rapid change in excitability, and refractoriness is reduced when the laser light is applied, potentially also ruling-out increased Na^+^ channel inactivation as the mechanism (Fig. 2E, F). This is because the primary mechanism involved in generating refractoriness, once nodal K^+^ channels are blocked, is the delayed escape of Na^+^ channels from inactivation (also known as repriming), and this mechanism is undoubtedly accelerated by the laser.

### Recording extracellular membrane potential indicates the change in membrane potential occurs over a minute

While it is not possible to record membrane potential in individual axons in the optic nerve, it has proved possible to record a fraction of the membrane potential of all the axons extracellularly eg^1^, across the grease barrier in the nerve bath. There are advantages associated with this approach. First, it is not necessary to damage the axons at the site of recording, and secondly the technique will pick up changes in potential even when some of the axons cannot be electrically stimulated further down the nerve, and so cannot contribute to a threshold measurement. However, the technique cannot provide a definitive value of membrane potential, and the size of the membrane potential changes observed depends on the quality of the seal at the barrier, that can change even within a single experiment. Nevertheless, where the seal resistance is high, mV changes in DC potential can be recorded across the barrier, that appear to be around half the change in potential across the membrane of an individual axon. Finally, it is possible to compare the effects of applying laser light and fluctuations in ambient bath-chamber temperature at closely similar times and in the same recordings (Fig. 3B).

### Superfusion of ZD7288 increases the change in threshold, consistent with IR laser light hyperpolarizing axons

**Figure 4:**
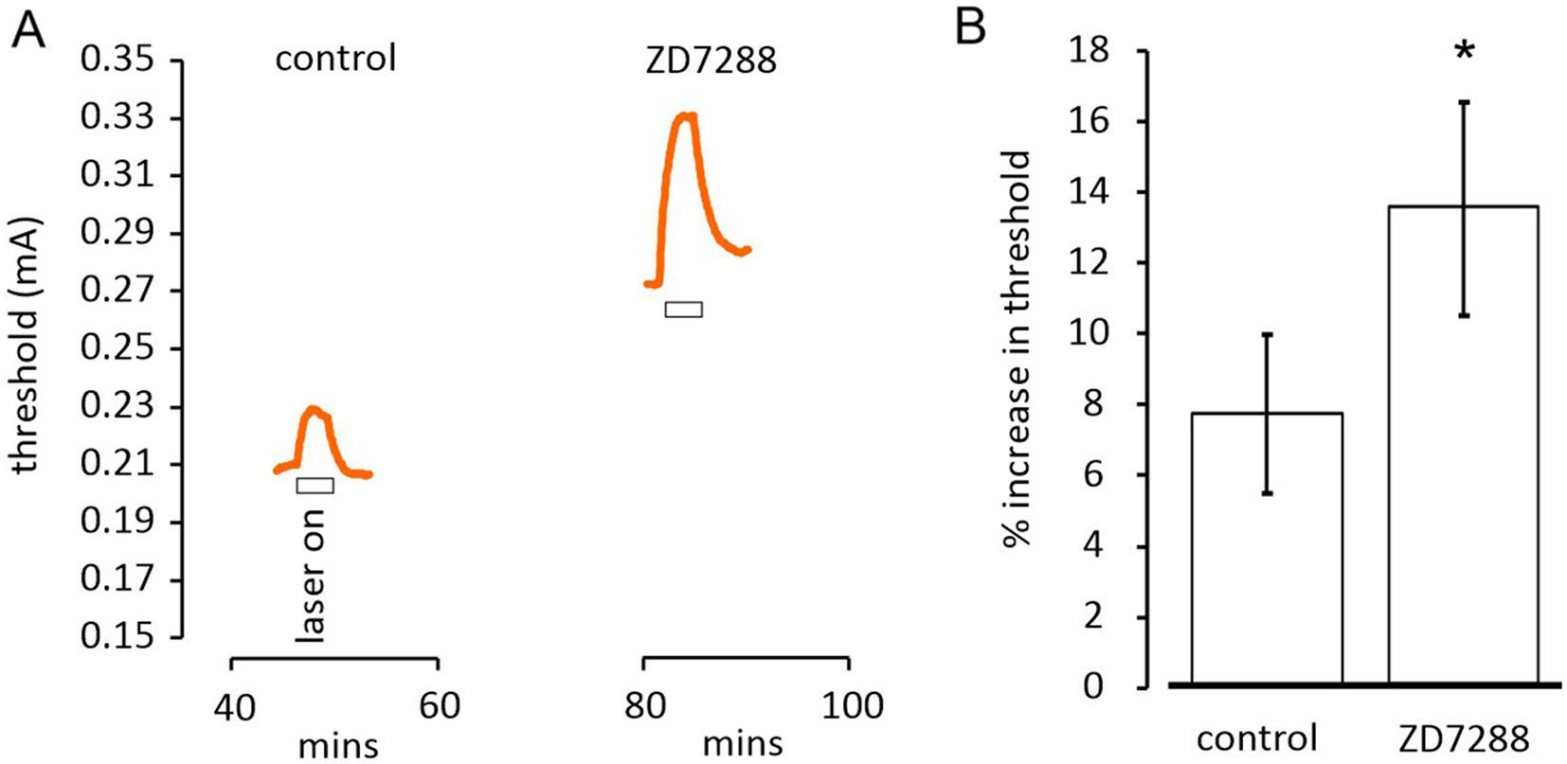
Pharmacological interaction with the effect of laser light by blocking *I*_h_. (**A**) Application of laser light (using 350 mA driving current) near the site of stimulation gives rise to an increase in threshold (as also shown in panel (**B**) control values). Exposure to ZD7288 for 30 min gives rise to an increase in resting threshold, because blocking *I*_h_ produces a resting hyperpolarization in the axons, and the increase in threshold caused by laser light becomes larger after the superfusion of 50 µM ZD7288, (**A**, **B**) (*P* = 0.034, paired t-test, *n* = 6). The effect is explained by a fall in the conductance of the axons and an increase in the slope of the temperature *versus* membrane potential relation (see “Discussion” section).

When the IR light is applied to the site of stimulation, threshold increases, and this is usually sustained over a 3 min application period (Fig. 3A). In order to help prove that IR light hyperpolarizes the axons using a pharmacological approach, we recorded the threshold-increases before and after superfusion of 50 µM ZD7288, a drug that blocks *I*_h_^13^. *I*_h_ is generated by HCN channels that are activated by hyperpolarization^8,14^. *I*_h_ should be able to supress a temperature rise-dependent hyperpolarization, so blocking the channels would be predicted to increase the hyperpolarization, and therefore increase the change in threshold brought about by laser light. We have found that ZD7288 increased the threshold-rise by 85.11 ± 28.19%, (*P* = 0.034, paired t-test, *n* = 6), although the responses were variable, Fig. 4. We present a mathematical treatment of this effect and explain how this is manifest. Further, the addition of ZD7288 to the perfusion solution gives rise to an increase in the resting threshold, an effect already reported in Austerschmidt et al.^3^, and we think this means the axon resting membrane potential hyperpolarizes with the loss of *I*_h_. This is expected to increase the resting potential by − 5 mV, or perhaps more, explained in the section immediately below.

### Explaining the effect of blocking *I*_h_

**Figure 5:**
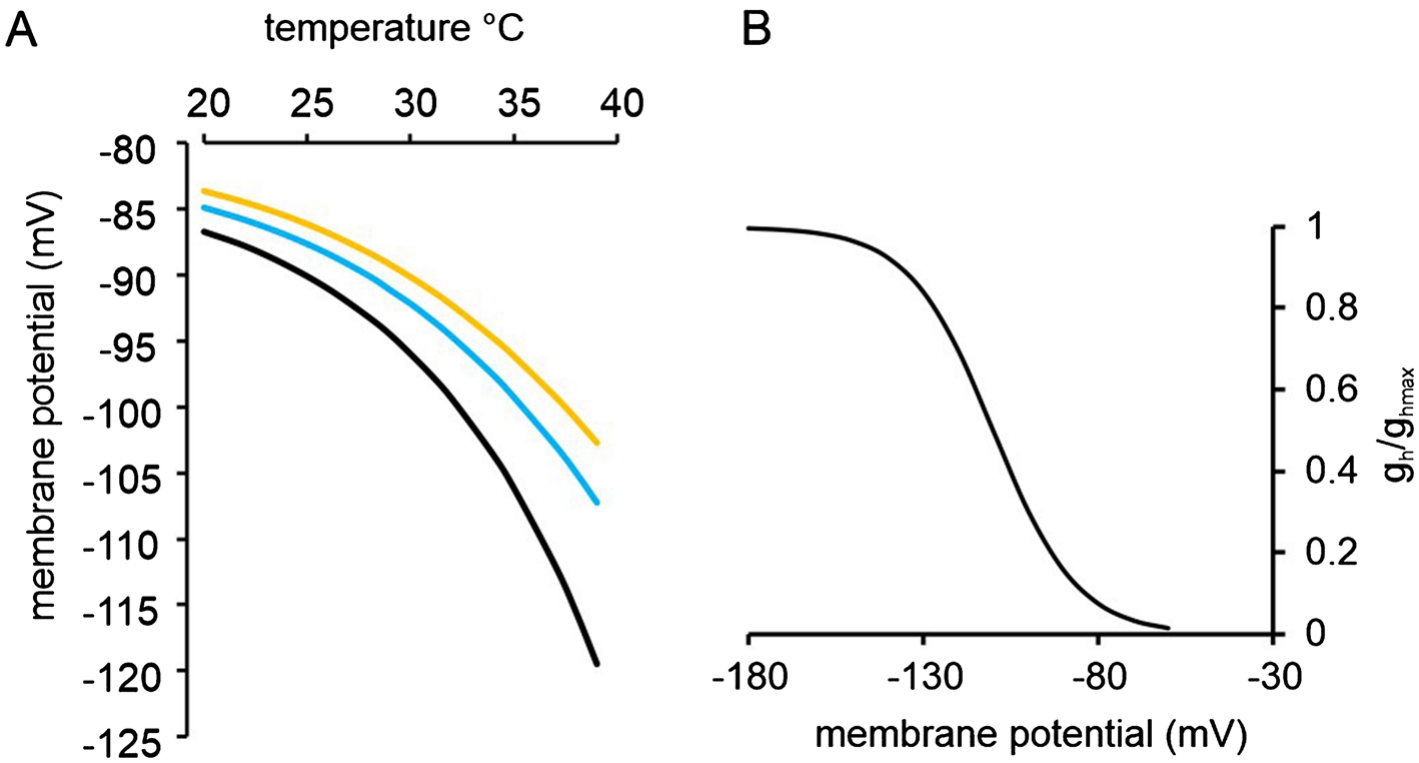
Changes in membrane potential associated with a temperature-dependent electroneutral influx of Na^+^ ions, is modified by *I*_h_. (**A**) where *I*_EN_, the return pump-current associated with the electroneutral influx, is set to 8 pA at 20 °C and increasing temperature increases electroneutral entry, with an assumed *Q*_10_ of 3 (without *I*_h_, black curve). Maximal activation of *I*_h_ increases membrane conductance by 100% (yellow curve), or by 50% (blue curve). (**B**) *I*_h_ activation curve plotted against membrane potential. As the membrane potential hyperpolarizes, Ih is activated more.

Equation (1) allows the estimation of the change in resting membrane potential when the temperature increases, by the expedient of allowing electroneutral Na^+^ entry to have a temperature dependent rate^3^. The equation incorporates resting Na^+^ and K^+^ conductances, in the simplest case neither of which exhibit voltage-dependence, a Na^+^-pump current returning both the electroneutral influx of Na^+^. This approach assumes that the intra and extra-axonal concentrations of Na^+^ and K^+^ are unchanging throughout. It is also assumed that the total resting membrane conductance is the sum of the Na^+^ and K^+^ conductances, independent of the contribution of individual channel subtypes such as voltage-gated Na^+^ channels and *I*_h_. Modifying the *g*_Na_ and *g*_K_ variables to allow an increase in membrane conductance on hyperpolarization, according to the voltage-dependence of *I*_h_ detailed in Mayer and Westbrook^14^, shows how the intrinsic temperature dependence of the resting membrane potential is predicted to be severely limited by the presence of *I*_h_ (Fig. 5, below).

At the resting potential there is no net current across the membrane. Therefore with a resting Na^+^ and K^+^ conductance and a parallel expulsion of Na^+^ entering electroneutrally:

1 $$g_{{{\text{Na}}}} \left( {E_{{\text{m}}} - E_{{{\text{Na}}}} } \right) + g_{{\text{K}}} \left( {E_{{\text{m}}} - E_{{\text{K}}} } \right) + I_{{{\text{EN}}}} = 0$$

where *g*_K_ is the membrane conductance for K^+^ ions, *E*_K_ the K^+^ ion equilibrium potential, *g*_Na_ is the membrane conductance for Na^+^ ions, *E*_Na_ is the Na^+^ ion equilibrium potential. *I*_EN_ is the pump current resulting from the electroneutral movement of Na^+^ across the membrane, and *E*_m_ is the resting membrane potential.

Allowing for the Na^+^ pump to return a current one third of any inward Na^+^ ionic current

2 $${\raise0.7ex\hbox{$2$} \!\mathord{\left/ {\vphantom {2 3}}\right.\kern-\nulldelimiterspace} \!\lower0.7ex\hbox{$3$}}g_{{{\text{Na}}}} \left( {E_{{\text{m}}} - E_{{{\text{Na}}}} } \right) + g_{{\text{K}}} \left( {E_{{\text{m}}} - E_{{\text{K}}} } \right) + I_{{{\text{EN}}}} = 0$$

Given the above, the following rearrangement applies:

3 $$E_{{\text{m}}} = {{\left( {g_{{\text{K}}} E_{{\text{K}}} + \left( {{\raise0.7ex\hbox{$2$} \!\mathord{\left/ {\vphantom {2 3}}\right.\kern-\nulldelimiterspace} \!\lower0.7ex\hbox{$3$}}} \right)g_{{{\text{Na}}}} E_{{{\text{Na}}}} - I_{{{\text{EN}}}} } \right)} \mathord{\left/ {\vphantom {{\left( {g_{{\text{K}}} E_{{\text{K}}} + \left( {{\raise0.7ex\hbox{$2$} \!\mathord{\left/ {\vphantom {2 3}}\right.\kern-\nulldelimiterspace} \!\lower0.7ex\hbox{$3$}}} \right)g_{{{\text{Na}}}} E_{{{\text{Na}}}} - I_{{{\text{EN}}}} } \right)} {\left( {\left( {{\raise0.7ex\hbox{$2$} \!\mathord{\left/ {\vphantom {2 3}}\right.\kern-\nulldelimiterspace} \!\lower0.7ex\hbox{$3$}}} \right)g_{{{\text{Na}}}} + g_{{\text{K}}} } \right)}}} \right. \kern-\nulldelimiterspace} {\left( {\left( {{\raise0.7ex\hbox{$2$} \!\mathord{\left/ {\vphantom {2 3}}\right.\kern-\nulldelimiterspace} \!\lower0.7ex\hbox{$3$}}} \right)g_{{{\text{Na}}}} + g_{{\text{K}}} } \right)}}$$

Two eventualities are apparent. The first is that if the electroneutral transmembrane Na^+^ flux and Na^+^ conductance are reduced to zero, the membrane potential (*E*_m_) becomes equal to *E*_K_, and the value of *E*_K_ is shown to be the most negative value possible for *E*_m_. The second is that with the introduction of electroneutral Na^+^ flux, it becomes possible for *E*_m_ to be more negative than *E*_K_.

Austerschmidt et al.^3^ used a membrane resistance of 0.55 GΩ for a length of optic nerve axon, the same value previously chosen by Harris and Attwell^15^. This corresponds to a transmembrane conductance of 1.82 nS, and where this conductance comprises the sum of *g*_Na_ = 0.29 nS and *g*_K_ = 1.53 nS, the resting membrane potential, without the effects of electroneutral Na^+^ entry, is − 82 mV, according to Eq. (3), where *E*_K_ = − 100 mV and *E*_Na_ is + 60 mV. With these same variable values, the effect of the Na^+^ pump on *E*_m_, in the absence of any electroneutral entry, is − 7.5 mV, and this component is not dependent on temperature, unless the value of *g*_Na_ alters. However, adding an electroneutral flux of Na^+^ of 2.0 × 10^−16^ mol/s increases *E*_m_ to − 85.8 mV, and where this is assumed to be occurring at 20 °C, an increase in temperature from 22 to 37 °C with a *Q*_10_ of 3 gives a hyperpolarization of − 19.8 mV.

Our experiments predict *I*_h_ must act to reduce the magnitude of hyperpolarization with warming (Fig. 5).

Adding a voltage-dependent *I*_h_, activating according to the relation shown in B, necessitated solving Eq. 3 numerically using a recursive program in MatLab. The maximally activated *g*_h_ was taken to increase the nerve membrane conductance by 100% or by 50% (orange and blue curves, respectively). *I*_h_ was taken to be the sum of ionic currents provided by a parallel voltage-dependent Na^+^ conductance and K^+^ conductance, with an overall reversal potential of − 36 mV^14^, and with a steady-state activation curve of this form:

4 $$g_{{\text{h}}} {/}g_{{{\text{hmax}}}} = 1{/}\left( {1 + \exp \left( {E_{{\text{m}}} - V_{0} } \right){/}k} \right)$$

where *g*_h_ is the conductance at any membrane potential expressed as a fraction of the maximal conductance, *E*_m_ is the membrane potential, *V*_0_ is the midpoint (− 110 mV) and *k* is the slope of the relation (taken as − 12 mV), cf^3,16^. A summary of the arrangement of ion channels and the Na^+^-pump in myelinated axons is available in the literature for peripheral nerve axons (^17^ ‘the Bostock model’), that must differ from the F-fibres in optic nerve only by degree (explored in^3^, and where the functionality of K_V_7 in optic nerve axons appears minimal). The major ionic leakage, electroneutral transport, pump, and *I*_h_ that concerns the present analysis are suggested to be found in internodal membrane, where voltage-dependent gating affects only *I*_h_, and is based only on steady-state conditions.

The presence of *I*_h_ depolarizes the nerve membrane, and more *I*_h_ is associated with a reduced slope of the membrane potential temperature-dependence. Exploring a scenario with variables similar to those thought to be applying in our experiments, where local temperature is raised 3 °C by applying laser light from an ambient bath temperature of 32 °C (and where a *g*_hmax_ is incorporated that increases the resting membrane conductance by 100% when fully activated), blocking *I*_h_ will increase the laser-induced change in membrane potential by 63.2%. This explains why the exposure to ZD7288 makes the threshold increases bigger. While this result is within a single standard error of the proportionally larger threshold increases, these increases, before and after exposure to ZD7288, may also be affected by differing degrees of resting Na^+^ channel inactivation with the pre-exposure effect being moderated by a greater removal of Na^+^ channel inactivation throughout the 3 min long exposure to the light.

### Ambient bath temperature affects the threshold increase caused by IR laser light

**Figure 6:**
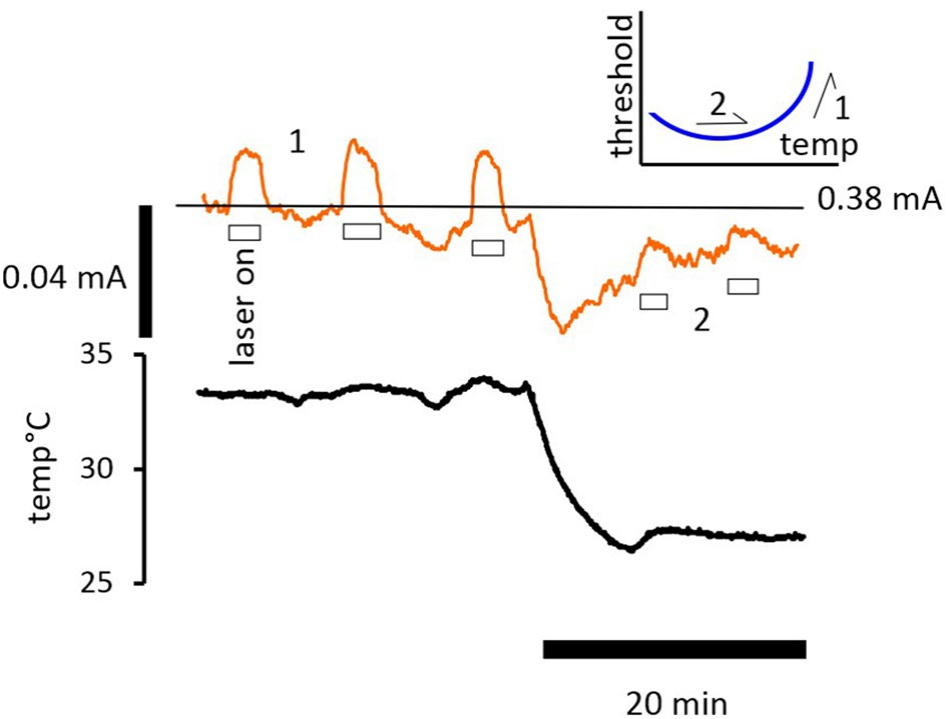
The increase in threshold brought about by laser light application depends on the ambient bath temperature. Application of laser light produces a larger effect on current threshold when the ambient bath temperature is near 33 °C, than at 26 °C. This observation complements the previously reported finding that the relationship between current threshold and temperature is a ‘U’ shaped one, with the minimum threshold being found near 28 °C ^2^. The threshold-temperature ‘U’ is indicated inset as a cartoon (blue curve). It is also consistent with a major part of the action of IR laser light being dependent on local warming within the nerve. Where the nerve is at 33 °C, the larger responses to laser light applications are indicated as state 1, and when at 26 °C, state 2, with more details given in the text. We suggest that the slow increase in threshold occurring after the fall in temperature in this experiment is likely caused by slow Na^+^ channel inactivation following the depolarization of the membrane potential with cooling.

Without altering either the position of the laser light guide, or changing the current driving the diode, our data suggest that threshold changes brought about by applying laser light depend on the bath temperature. This would make sense if the bath temperature had a role to play in setting the resting potential of the axons, which we have previously reported, and if the laser brought about changes in threshold by warming the nerve. Applying laser light when the nerve is at 26 °C and at 33 °C gives rise to different responses, as shown in Fig. 6, and these differences can be explained easily as the relationship between threshold and temperature is ‘U’ shaped^2^. In the case where the bath temperature is near 26 °C, laser light induced warming of a few single degrees centigrade takes the nerve across the bottom of the ‘U’, indicated as 2 on the inset cartoon, whereas at a warmer bath temperature, the threshold increases more because the nerve is made to ascend the right hand side of the ‘U’, indicated as 1. One possibility is that the small threshold-changes seen at 26 °C equate to the fast changes only. The nerve was exposed to 5 µM bumetanide throughout this recording, revealing that although a part of the electroneutral Na^+^ flux was blocked, it is not sufficiently reduced to prevent temperature dependent changes in threshold, nor all the effects of IR laser light. Our previous findings indicate that bumetanide will reduce electroneutral Na^+^ entry, for example slowing the decline in the action potential amplitude in the presence of the Na^+^-pump blocker ouabain^2^, and blocking the transporter NKCC1 will cause a small (probably single mV) reduction in resting membrane potential as a result^3^. However, most electroneutral Na^+^ movement appears to be not sensitive to bumetanide.

## Discussion

The primary finding of these experiments is that it is possible to change optic nerve axon excitability, in a manner partly consistent with what has been found previously using whole-bath warming, using light from a commercially rated 5 mW near IR-laser diode.

The effects of energetic IR-laser light on cell membranes has been reported previously eg^5^, and it is expected to be excitatory. The proposed mechanism is a reversible increase in membrane capacitance, associated with capacity currents and the partial loss of the resting potential. The effects we report here are very different, brought about by a laser with over an order of magnitude less power and associated with at least two mechanisms of reduced excitability. Our findings also seem to suggest a possible block of action potential propagation in some myelinated units, explaining the fall in compound action potential amplitude. It is also evident that laser induced effects on threshold cannot be blocked by the electroneutral transport blocker bumetanide^2^, that we know can partially antagonise the effects of raised bath perfusion temperature on threshold, and there are probably two reasons for this. First, the contribution to electroneutral Na^+^ transport by NKCC1 is probably far from being the major part. Our previous work suggests that bumetanide and the removal of extracellular Cl^-^ not only block a similar part of the electroneutral Na^+^ flux, but that this movement probably only adds single millivolts to the membrane potential^3^. Secondly, if we are right that the rapid effects of the laser cannot be caused by Na^+^ movements (that would be expected to lag behind a temperature increase) then there must be another effect, different in kind, that explains it. We suggest altered myelin function, and this is discussed further, below.

In our recordings, the activity of 4-AP in the nerve is shown by the clear effects on F-fibre action potential width, and DAP amplitude. These findings can be explained by the presence of kinetically fast K^+^ channels at F-fibre nodes of Ranvier, that must normally contribute to action potential termination and that may correspond to those immunohistochemically identified as K_V_3.1b^11,18^. Further, as in the periphery, internodal K^+^ channels controlling the DAP must also be blocked by 4-AP eg^9,19^.

Following local light irradiation, the evidence for a rapid change in axonal function (happening within single seconds) includes the immediate fall in the tracked response amplitude, and the change in compound action potential kinetics (Fig. 1), that are apparent within one or two cycles of the threshold-tracking protocol. We suggest these rapid changes are occurring because of rapid local changes in nerve temperature. In addition to this we have measured rapid increases in threshold in the largest fibres, using threshold-tracking, that are not sustained over minutes (Fig. 2E), and that also exist when the nerve is close to the bottom of our previously defined threshold-temperature ‘U’, highly suggestive that these rapid changes are related to the laser light application and are not the same as the effects of changing the bath temperature only (Fig. 6). The transient increase in threshold recorded in this situation is also much larger in magnitude than could be predicted for a few single degrees centigrade increase in bath temperature (Fig. 2E). From our experiments with 200 µM 4-AP, we have argued it is unlikely that following irradiation a change in kinetically fast K^+^ channel function, and, or an increase in Na^+^ channel inactivation are able to explain this phenomenon. Instead, we hypothesize that an alteration in myelin function (perhaps with reversible expansion or lifting of the sheath), causing a fall in transmyelin (Barrett and Barrett) resistance is a candidate that would explain a rapid fall in excitability. We therefore propose that the rapid effects of the laser are caused by local nerve temperature increases, precipitating immediate changes to action potential kinetics and perhaps myelin function. The slower changes in threshold seem adequately explained by a membrane potential hyperpolarization, and if this is in fact the case, there is probably little difference between these slower effects of the laser and the effect of increasing the temperature of the whole nerve bath. We suggest the time-course of increasing superexcitability and falling refractoriness (Fig. 2B, Supplementary Fig. 1) likely parallel the membrane potential change. The pharmacological action of ZD7288 that we report confirms that a hyperpolarization has occurred over 3 min, and that this hyperpolarization is enhanced by blocking *I*_h_. Finally we hypothesize this longer time-course of hyperpolarization would be consistent with a requirement for ion transport before the membrane potential changes. There is little doubt that there must be rapid changes in threshold when the IR laser light is applied to the nerve, and we have hypothesized that this could be caused by a fall in the Barrett and Barrett resistance across the myelin. However, such a scenario may also help explain the apparently rapid changes in DAP amplitude reported in Fig. 1, because a fall in transmyelin resistance should also allow more rapid charging of the internodal axolemma from active nodes. An increase in the DAP amplitude during laser light irradiation might therefore be explained by some combination of a fall in transmyelin resistance (suggested to be rapid) and a more gradual membrane potential hyperpolarization.

We have previously reported the relationship between current-threshold and temperature is ‘U’-shaped, the right hand side of the ‘U’ being dominated by the change in membrane potential associated with changes in temperature. The bottom of the ‘U’ and the left-hand side, are importantly impacted by the availability and slowed activation kinetics of Na^+^ channels, as channel inactivation is expected to increase when the temperature is lowered down to and through 28 °C^2,12^. Further evidence that the laser light application works through local temperature increases is to be found from the observation that the increase in threshold with light is much reduced with prolonged irradiation when the ambient bath temperature is at 26 °C, in comparison to a warmer temperature (Fig. 6).

The application of prolonged polarizing currents is well understood to produce changes in nerve excitability^12,20^. For example, a hyperpolarizing current applied at a point generates a negative going electrotonic change in membrane potential, affecting nearby nodes and internodes and moving the membrane potential away from firing threshold. So long as the nerve is not depolarized *before* a hyperpolarizing current is applied (a state associated with significant resting Na^+^ channel inactivation), the threshold changes evolve over time in a manner paralleling the changes in the electrotonic potential, giving threshold-electrotonus TE^12^. During the application of IR laser light to nerve we have found that larger stimulus currents are required to excite axons at the locality, and circumscribed warming using laser light must move the local membrane potential away from firing threshold by producing a persistent hyperpolarization (that might be called photo-electrotonus PE), and also a persistent increase in threshold. Unlike the application of current from an external electrode, that will differentially polarize nodes and internodes because of the presence of the Barrett and Barrett resistance across the myelin^7,8^, our explanation of the phenomenon means that PE is dependent on a Na^+^ pump induced hyperpolarization. This is expected to occur along the whole axon membrane and where most Na^+^/K^+^-ATPase expression is internodal eg^21^. The transmembrane nodal potential will be the same as that at the internode, so long as the net current across the internodal membrane is zero.

## Methods

### Animals

Animals were housed in a Home Office designated establishment at Barts and the London medical school, with food and water ad-libitum, under veterinary supervised conditions. As reported previously eg^3^, adult male Wistar rats and female Sprague Dawley rats (~ 320 g), obtained from an approved supplier, underwent euthanasia by exposure to a rising concentration of CO_2_ followed by cervical dislocation, as specified by the detailed guidance in the Schedule 1 protocol list and in accordance with UK legislation (and this includes ethical permission), published by the Home Office UK (Scientific Procedures Act 1986), before tissues were removed.

### Electrophysiology

Details of the recording bath have been published previously eg^1^, and a simplified diagram is shown in Supplementary Figure S2. The rat optic nerves, with eyeballs attached, were isolated and placed in oxygenated buffer. Once any tissues adherent to the nerve and eyeball were removed, the nerve was mounted in a two-chambered nerve bath, using a venous cannula to position the nerve across the recording barrier. The barrier separated two chambers filled with buffer solution. The right hand chamber allowed for stimulation of the nerve (using a suction electrode), and was perfused with oxygenated buffer solution whose temperature could be made to vary. This solution flow was also used to superfuse drugs across the whole of the nerve in the chamber. Temperature control in the chamber was achieved using a heating wand (HPT-2 ALA scientific, Farmingdale, NY, USA), with a thermistor placed near the nerve (TC10 npi electronic, Tamm, Germany), allowing measurement of the ambient bath temperature. The left hand chamber housed the eyeball, and membrane potential recordings could be made across the grease-gap at the barrier, where recording electrodes were placed in both the left and right hand chamber.

We recorded the action potential responses to supra-maximal stimuli, and also used the threshold-tracking technique to investigate changes in electrical threshold in the right hand chamber, at the bath end of the suction electrode. The nerve was stimulated using 200 µs constant-current pulses generated by a computer controlled stimulator (Digitimer DS4, Welwyn Garden City, Herts, UK). Control of stimulus current amplitude was achieved using a PC running QTRAC-S (Hugh Bostock, Institute of Neurology; available through Digitimer). For most recordings of this type (with the exception of data shown in Figs. 2 and 6), the stimulus amplitude was maintained close to 50% of the supramaximal response, and this corresponded to an action potential mainly, although not wholly, attributable to the F-fibres in the nerve, representing axons with the highest conduction velocity and that are likely to be the largest. Otherwise a 5% response was set as the target amplitude. This ensured that the F-fibres alone were excited. Laser light was applied locally, at the site of stimulation or recording, by manoeuvring the end of the optic fibre light guide to the appropriate location in the right hand chamber of the bath. Recordings of action potentials and depolarizing after potentials were made AC, with high-pass filtered at 0.1 Hz, whereas recordings of slow changes in membrane potential were made without high-pass filtering (ie DC).

Superexcitabilty was recorded when threshold-tracking, by providing a conditioning stimulus, 1.5 times the amplitude of that of the just previously measured test stimulus, and preceding the test stimulus by 10 ms. The responses following a conditioning stimulus were recorded in a separate channel in QTRAC-S from that of the control test stimulus responses, and these channels alternated in presentation, every 2 s. Superexcitability was therefore measured during the depolarizing afterpotential that followed the axonal activation in response to the conditioning stimulus. Similarly, refractoriness was measured with a shorter 4 ms delay following the conditioning stimulus, during the relative-refractory period.

### Laser diode

The laser diode used had an integral fibre-optic (PL15D005100A-0-0-01; Laser components (UK) Ltd, Chelmsford, Essex, UK), that could be made to deliver light energy to certain locations along the optic nerve. The fibre-optic diameter was 9 µm. The two locations targeted in these experiments were the site of stimulation, at the end of the stimulating electrode, and the site of recording, close to the grease barrier in the right hand chamber. The laser was commercially rated as 5 mW, and generated IR light at 1550 nm. The diode was completely enclosed, apart from the fibre-optic output, and was driven from a constant-current bench power supply (Thurlby-Thandar id, Huntingdon, Cambs UK), using continuous currents up to 400 mA, that were adjusted during an experiment. Our experimental data indicate that operating the laser diode continuously over 2 or 3 min did not produce a substantial fade in output, and the continuous output with 400 mA was empirically estimated as close to 10 mW from the measured photo-thermal effect in 100 µl of water. The arrangement of the bath with laser light irradiation sites is shown in Figure S2, and bath perfusion temperature was maintained close to 32 °C unless explicitly stated.

### Solutions

Control buffer solution contained (in mM): NaCl 140, HEPES hemi Na 10, CaCl_2_ 2.1, MgCl_2_ 2.12, KCl 2.5, Glucose 10. It was adjusted to pH 7.2–7.3 with the addition of small quantities of HCl, as necessary. 4-Ethylphenylamino-1,2-dimethyl-6-methylaminopyrimidinium chloride (ZD7288) and bumetanide were obtained from Tocris (Bio-techne Ltd, Abingdon, Oxfordshire, UK). These drugs were made up as stock solutions in DMSO, at 50 mM and 5 mM, respectively, and stored at − 20 °C. The final concentrations were achieved by one-thousand fold dilution and the concentration of vehicle in the nerve bath never exceeded 0.1%. 4-Aminopyridine (4-AP) was obtained from Sigma-Aldrich (Merck, Poole, Dorset, UK), and was made as a fresh solution from the crystalline form on each experimental day, ensuring appropriate pHing of the buffer solution took place using HCl.

### Statistical analysis

Statistical analysis was undertaken using paired-t test, or single-sample t-test, with the critical value of *P* taken as 0.05. Experimental values are quoted or plotted as means ± SEM wherever possible.

## Supplementary Information

Supplementary Figures.

## Data Availability

Any data generated during or analysed for the study reported here are available from the corresponding author at reasonable request.
