# Supplementary material for: Changing the firing threshold for normal optic nerve axons by the application of infra-red laser light
Source: Sci Rep. 2021 Oct 15;11:20528. doi: 10.1038/s41598-021-00084-1 (PMC8519963; doi:10.1038/s41598-021-00084-1)
Supplement: Supplementary file 1 — Supplementary Figures. [file 41598_2021_84_MOESM1_ESM.pdf]

Changing the firing threshold for normal optic nerve axons by the application of infra-red laser light

Lavinia J Austerschmidt, Nadine I Schottler, Alyssa M Miller and Mark D Baker

Centre for Neuroscience, Surgery and Trauma, Blizard Institute, QMUL 4 Newark Street Whitechapel, London E1 2AT, UK.

Supplementary figure S1

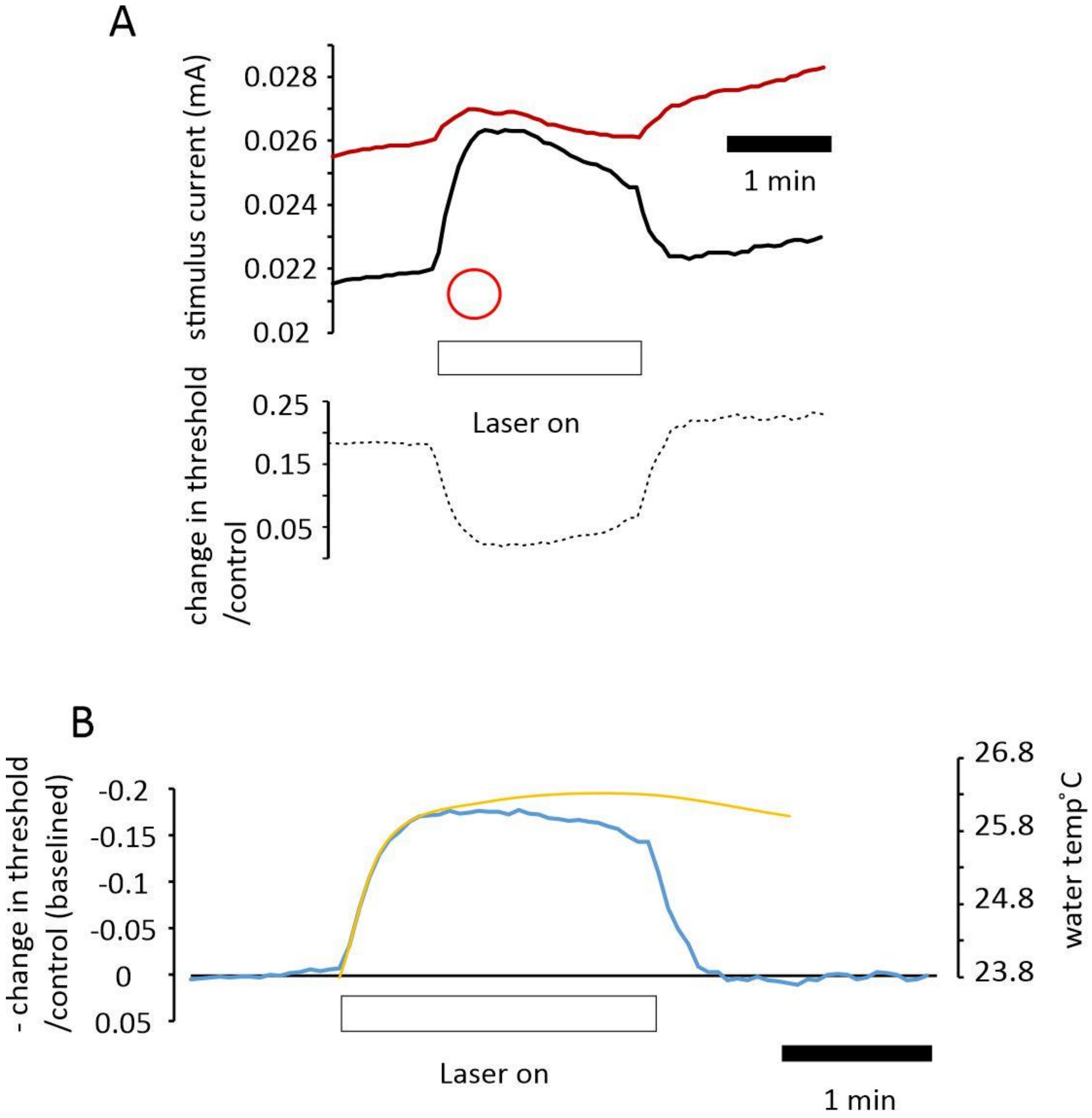

Figure S1. The rapid effect of the laser increases threshold for F-fibre responses in the control, and also after a conditioning stimulus at 4 ms, with 200 μM 4-AP throughout. A, upper panel, control threshold (black-trace) shows the rapid effect of the laser (400 mA driving current, approximate timing indicated by red circle) with an increase too large to be accommodated by the already reported threshold/temperature relation. The increase in threshold is also seen in the refractory condition (red trace), where an increase in temperature or membrane potential might be expected to cause a fall in refractoriness. The rapid effect on control-threshold appears to wane, leaving a sustained raised threshold at 2 mins. Lower panel, refractoriness at 4 ms expressed as a fractional change in threshold, relative to the control. The data reveal a fall in refractoriness at the same time as threshold increases with laser light application. B, The relative change in refractoriness (blue, laser for 2 mins) has a very similar trajectory as the temperature change measured at the end of the light guide in 2 mls distilled water (orange), following light application (3 mins).

Supplementary figure S2

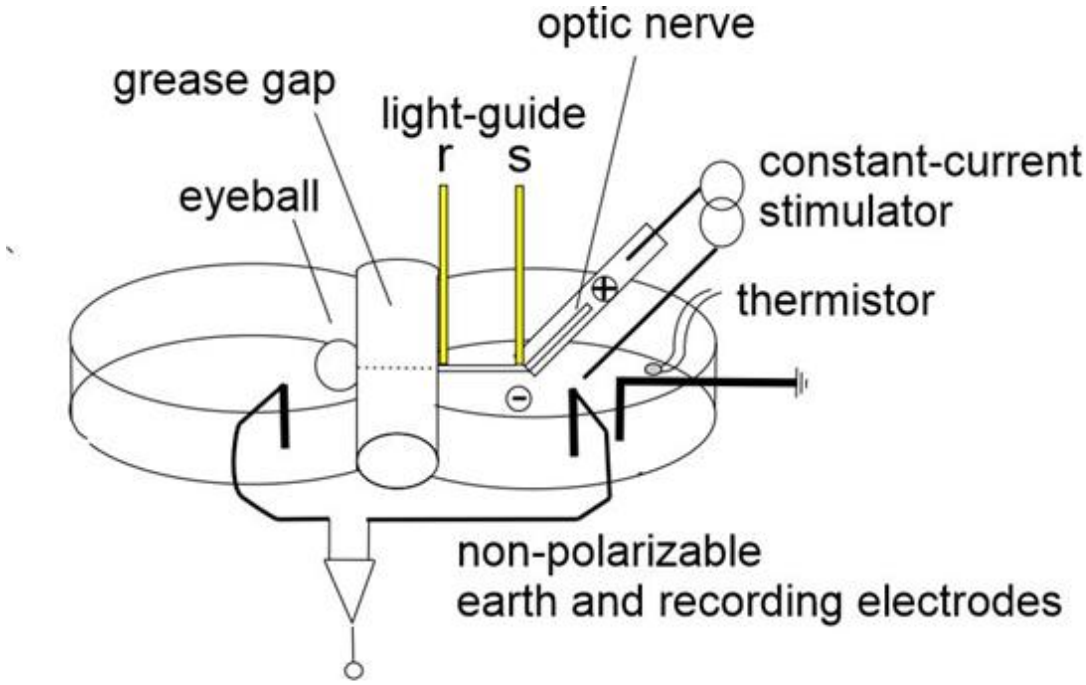

Figure S2. Diagram of optic nerve stimulating and recording arrangement, with laser light-guides. The optic nerve was mounted across a grease gap between two modified 35 mm Petri dishes. The light guides contacted the optic nerve at the site of recording, next to the grease gap (r), or at the site of stimulation (s). The fibre optic was 9 μm in diameter and embedded in a flexible plastic sheath. Light irradiation had no effects on measured bath temperature, and no effects on nerve function unless the guides were in physical contact with the nerve.
